# Supplementary material for: Hot spot prediction in protein-protein interactions by an ensemble system
Source: BMC Syst Biol. 2018 Dec 31;12(Suppl 9):132. doi: 10.1186/s12918-018-0665-8 (PMC6311905; doi:10.1186/s12918-018-0665-8)
Supplement: Supplementary file 2 — All relevant MATLAB code. A simple Matlab implement of our predictor. (ZIP 19,742 kb) [file 12918_2018_665_MOESM2_ESM.zip › Additional file 2/Read ASA/ASA website/SKEMPI/2WPTB.htm]

 NetSurfP - Protein Surface Accessibility and Secondary Structure Predictions

  

|  |  |  |
| --- | --- | --- |
|  |  | NetSurfP - Protein Surface Accessibility and Secondary Structure Predictions  Technical University of Denmark |

  


---

```
# For publication of results, please cite:
# A generic method for assignment of reliability scores applied to solvent accessibility predictions.
# Bent Petersen, Thomas Nordahl Petersen, Pernille Andersen, Morten Nielsen and Claus Lundegaard
# BMC Structural Biology 2009, 9:51 doi:10.1186/1472-6807-9-51
#
# Column 1: Class assignment - B for buried or E for Exposed - Threshold: 25% exposure, but not based on RSA
# Column 2: Amino acid
# Column 3: Sequence name
# Column 4: Amino acid number
# Column 5: Relative Surface Accessibility - RSA
# Column 6: Absolute Surface Accessibility
# Column 7: Z-fit score for RSA prediction
# Column 8: Probability for Alpha-Helix
# Column 9: Probability for Beta-strand
# Column 10: Probability for Coil
E P  2WPTB                  1    0.703  99.770  -0.642   0.003   0.003   0.994
E G  2WPTB                  2    0.482  37.918  -2.931   0.004   0.197   0.799
E K  2WPTB                  3    0.502 103.303  -0.436   0.004   0.514   0.481
B A  2WPTB                  4    0.235  25.941  -2.296   0.004   0.514   0.481
E T  2WPTB                  5    0.415  57.533  -0.680   0.022   0.359   0.619
B G  2WPTB                  6    0.282  22.170  -2.130   0.022   0.359   0.619
E K  2WPTB                  7    0.506 104.002   0.410   0.021   0.279   0.699
B G  2WPTB                  8    0.312  24.578  -1.432   0.020   0.205   0.775
E K  2WPTB                  9    0.425  87.340   0.874   0.022   0.359   0.619
E P  2WPTB                 10    0.563  79.876   0.267   0.021   0.451   0.528
B V  2WPTB                 11    0.108  16.569   0.138   0.069   0.386   0.545
E G  2WPTB                 12    0.359  28.230  -1.063   0.125   0.227   0.648
E D  2WPTB                 13    0.367  52.899  -1.419   0.191   0.086   0.723
E K  2WPTB                 14    0.373  76.644   0.370   0.321   0.252   0.427
B W  2WPTB                 15    0.106  25.421   0.195   0.428   0.171   0.402
B L  2WPTB                 16    0.160  29.278   0.643   0.605   0.105   0.290
E D  2WPTB                 17    0.407  58.620  -1.058   0.455   0.046   0.498
B D  2WPTB                 18    0.318  45.795  -1.816   0.430   0.016   0.555
B A  2WPTB                 19    0.257  28.354  -1.298   0.257   0.016   0.727
B G  2WPTB                 20    0.290  22.823  -1.680   0.181   0.016   0.803
E K  2WPTB                 21    0.547 112.518   0.031   0.115   0.016   0.868
E D  2WPTB                 22    0.473  68.159  -2.534   0.058   0.017   0.925
E S  2WPTB                 23    0.404  47.325  -1.434   0.018   0.019   0.964
E G  2WPTB                 24    0.300  23.641  -1.846   0.018   0.047   0.935
B A  2WPTB                 25    0.134  14.712  -0.125   0.019   0.141   0.840
B P  2WPTB                 26    0.266  37.745  -0.705   0.056   0.142   0.802
B I  2WPTB                 27    0.095  17.556   0.979   0.113   0.087   0.800
B P  2WPTB                 28    0.084  11.863   0.073   0.561   0.047   0.393
E D  2WPTB                 29    0.324  46.660   0.134   0.858   0.002   0.139
E R  2WPTB                 30    0.358  82.028   0.731   0.879   0.010   0.111
B I  2WPTB                 31    0.036   6.734   0.087   0.879   0.010   0.111
B A  2WPTB                 32    0.078   8.585  -0.041   0.879   0.010   0.111
E D  2WPTB                 33    0.421  60.709  -0.105   0.802   0.014   0.185
E K  2WPTB                 34    0.367  75.554   1.172   0.802   0.014   0.185
B L  2WPTB                 35    0.078  14.355  -0.624   0.622   0.015   0.363
E R  2WPTB                 36    0.382  87.478   0.272   0.339   0.016   0.645
E D  2WPTB                 37    0.562  80.984  -0.866   0.181   0.016   0.803
E K  2WPTB                 38    0.419  86.127   0.891   0.113   0.043   0.844
E E  2WPTB                 39    0.533  93.115   0.709   0.113   0.087   0.800
B F  2WPTB                 40    0.113  22.679   0.095   0.053   0.043   0.903
E K  2WPTB                 41    0.523 107.519  -0.190   0.058   0.017   0.925
E S  2WPTB                 42    0.323  37.820  -0.302   0.018   0.019   0.964
B F  2WPTB                 43    0.113  22.739   0.509   0.406   0.004   0.590
E D  2WPTB                 44    0.489  70.450   0.245   0.694   0.003   0.303
E D  2WPTB                 45    0.433  62.453   0.819   0.782   0.003   0.216
B F  2WPTB                 46    0.049   9.915   0.424   0.858   0.002   0.139
B R  2WPTB                 47    0.097  22.167   0.700   0.923   0.002   0.076
E K  2WPTB                 48    0.478  98.263   1.072   0.970   0.001   0.030
E A  2WPTB                 49    0.241  26.602   0.872   0.970   0.001   0.030
B V  2WPTB                 50    0.040   6.102   0.486   0.970   0.001   0.030
B W  2WPTB                 51    0.066  15.897   0.542   0.970   0.001   0.030
E E  2WPTB                 52    0.369  64.394   1.850   0.970   0.001   0.030
B E  2WPTB                 53    0.159  27.795   0.291   0.938   0.007   0.055
B V  2WPTB                 54    0.045   6.840   0.972   0.802   0.014   0.185
B S  2WPTB                 55    0.230  26.968  -0.543   0.430   0.016   0.555
E K  2WPTB                 56    0.668 137.387   0.450   0.109   0.005   0.886
B D  2WPTB                 57    0.224  32.293   0.323   0.053   0.005   0.942
E P  2WPTB                 58    0.433  61.429  -0.073   0.600   0.003   0.397
E E  2WPTB                 59    0.576 100.575   0.512   0.802   0.014   0.185
B L  2WPTB                 60    0.086  15.747   0.606   0.802   0.014   0.185
B S  2WPTB                 61    0.094  10.958   0.117   0.802   0.014   0.185
E K  2WPTB                 62    0.610 125.456   0.900   0.717   0.014   0.269
E N  2WPTB                 63    0.314  45.911   0.220   0.622   0.015   0.363
B L  2WPTB                 64    0.061  11.096   0.386   0.321   0.003   0.675
E N  2WPTB                 65    0.341  49.966   0.532   0.109   0.005   0.886
E P  2WPTB                 66    0.655  92.987   0.393   0.430   0.016   0.555
E S  2WPTB                 67    0.456  53.431  -0.762   0.406   0.004   0.590
B N  2WPTB                 68    0.166  24.288  -1.422   0.321   0.003   0.675
E K  2WPTB                 69    0.272  55.930   0.417   0.717   0.014   0.269
E S  2WPTB                 70    0.503  58.952   0.347   0.717   0.014   0.269
B S  2WPTB                 71    0.212  24.893   0.793   0.751   0.050   0.199
B V  2WPTB                 72    0.085  13.018   0.108   0.717   0.014   0.269
E S  2WPTB                 73    0.500  58.577   0.253   0.660   0.049   0.291
E K  2WPTB                 74    0.591 121.569  -0.119   0.257   0.016   0.727
E G  2WPTB                 75    0.394  30.976  -1.809   0.058   0.017   0.925
E Y  2WPTB                 76    0.307  65.563  -0.663   0.018   0.019   0.964
B S  2WPTB                 77    0.188  21.998  -0.847   0.018   0.019   0.964
B P  2WPTB                 78    0.211  29.870  -1.756   0.018   0.047   0.935
E F  2WPTB                 79    0.362  72.573  -0.523   0.018   0.088   0.893
B T  2WPTB                 80    0.087  12.067  -0.933   0.018   0.047   0.935
E P  2WPTB                 81    0.329  46.671   0.432   0.018   0.047   0.935
E K  2WPTB                 82    0.477  98.181  -0.471   0.113   0.043   0.844
E N  2WPTB                 83    0.444  65.002  -1.384   0.113   0.043   0.844
E Q  2WPTB                 84    0.389  69.440  -0.887   0.118   0.150   0.732
E Q  2WPTB                 85    0.470  83.996   0.048   0.125   0.227   0.648
B V  2WPTB                 86    0.165  25.330  -1.416   0.064   0.216   0.721
E G  2WPTB                 87    0.403  31.732  -0.918   0.018   0.088   0.893
E C  2WPTB                 88    0.525  73.766  -0.671   0.018   0.088   0.893
E R  2WPTB                 89    0.367  83.997   0.169   0.005   0.262   0.733
E K  2WPTB                 90    0.439  90.405   0.395   0.004   0.514   0.481
E V  2WPTB                 91    0.427  65.584   1.676   0.003   0.718   0.279
B Y  2WPTB                 92    0.074  15.750   0.460   0.018   0.846   0.136
B E  2WPTB                 93    0.187  32.634   0.418   0.011   0.918   0.071
B L  2WPTB                 94    0.035   6.335   0.340   0.011   0.918   0.071
B H  2WPTB                 95    0.166  30.177   0.949   0.021   0.756   0.223
B H  2WPTB                 96    0.108  19.645   0.388   0.021   0.451   0.528
E D  2WPTB                 97    0.570  82.166   0.438   0.018   0.088   0.893
E K  2WPTB                 98    0.478  98.407  -1.384   0.018   0.047   0.935
B G  2WPTB                 99    0.215  16.936  -1.810   0.018   0.088   0.893
B E  2WPTB                100    0.197  34.416  -0.660   0.021   0.279   0.699
B V  2WPTB                101    0.091  13.925  -0.453   0.021   0.451   0.528
B Y  2WPTB                102    0.128  27.418   1.160   0.022   0.552   0.426
B D  2WPTB                103    0.214  30.823  -0.041   0.021   0.451   0.528
B M  2WPTB                104    0.071  14.187   0.484   0.021   0.279   0.699
E D  2WPTB                105    0.311  44.873  -0.621   0.019   0.141   0.840
B N  2WPTB                106    0.222  32.428  -0.531   0.020   0.205   0.775
B I  2WPTB                107    0.052   9.638   0.485   0.023   0.655   0.322
B R  2WPTB                108    0.235  53.907   0.850   0.018   0.846   0.136
B V  2WPTB                109    0.069  10.590  -0.568   0.018   0.846   0.136
B T  2WPTB                110    0.199  27.560  -0.350   0.023   0.655   0.322
B T  2WPTB                111    0.214  29.626   0.287   0.004   0.197   0.799
E P  2WPTB                112    0.361  51.297  -1.170   0.018   0.088   0.893
E K  2WPTB                113    0.816 167.892   0.340   0.000   0.000   0.999

Explain the output.  Go back.
```

---
